# Relationships Between Memory Impairments and Hippocampal Structure in Patients With Subcortical Ischemic Vascular Disease

**DOI:** 10.3389/fnagi.2022.823535

**Published:** 2022-04-18

**Authors:** Miao He, Yang Li, Lijing Zhou, Yajun Li, Ting Lei, Wei Yan, Jiarui Song, Li Chen

**Affiliations:** ^1^Department of Radiology, Affiliated Hospital of North Sichuan Medical College, Nanchong, China; ^2^Department of Radiology, Gaoping District People’s Hospital, Nanchong, China

**Keywords:** hippocampal subfields, memory dysfunction, cognitive impairment, magnetic resonance imaging, subcortical ischemic vascular disease

## Abstract

**Background and Purpose:**

Patients with subcortical ischemic vascular disease (SIVD) suffer from memory disorders that are thought to be associated with the hippocampus. We aimed to explore changes in hippocampal subfields and the relationship between different hippocampal subfield volumes and different types of memory dysfunction in SIVD patients.

**Methods:**

A total of 77 SIVD patients with cognitive impairment (SIVD-CI, *n* = 39) or normal cognition (HC-SIVD, *n* = 38) and 41 matched healthy controls (HCs) were included in this study. Memory function was measured in all subjects, and structural magnetic resonance imaging (MRI) was performed. Then, the hippocampus was segmented and measured by FreeSurfer 6.0 software. One-way ANOVA was used to compare the volume of hippocampal subfields among the three groups while controlling for age, sex, education and intracranial volume (ICV). Then, *post hoc* tests were used to evaluate differences between each pair of groups. Finally, correlations between significantly different hippocampal subfield volumes and memory scores were tested in SIVD patients.

**Results:**

Almost all hippocampal subfields were significantly different among the three groups except for the bilateral hippocampal fissure (*p* = 0.366, *p* = 0.086, respectively.) and left parasubiculum (*p* = 0.166). Furthermore, the SIVD-CI patients showed smaller volumes in the right subiculum (*p* < 0.001), CA1 (*p* = 0.002), presubiculum (*p* = 0.002) and molecular layer of the hippocampus (*p* = 0.017) than the HC-SIVD patients. In addition, right subiculum volumes were positively related to Rey’s Auditory Verbal Learning Test (RAVLT) word recognition (*r* = 0.230, *p* = 0.050), reverse digit span test (R-DST) (*r* = 0.326, *p* = 0.005) and Rey–Osterrieth Complex Figure Test (ROCF) immediate recall (*r* = 0.247, *p* = 0.035) scores, right CA1 volumes were positively correlated with RAVLT word recognition (*r* = 0.261, *p* = 0.026), and right presubiculum volumes showed positive relationships with R-DST (*r* = 0.254, *p* = 0.030) and ROCF immediate recall (*r* = 0.242, *p* = 0.039) scores.

**Conclusion:**

SIVD might lead to general reductions in volume in multiple hippocampal subfields. However, SIVD-CI patients showed atrophy in specific subfields, which might be associated with memory deficits.

## Introduction

Subcortical ischemic vascular disease (SIVD) is a major vascular disease leading to dementia, cognitive decline, and stroke. Its neuroimaging signatures include white matter hyperintensities (WMH), lacunar infarcts, microbleeds, and enlarged perivascular spaces (PVS) (Shi et al., 2018; Ter Telgte et al., 2018). Although the major cognitive impairment associated with SIVD is considered to be executive dysfunction, memory decline has also been observed (Wang et al., 2010) and is known to be impaired early in cognitive impairment (CI) stage (Caillaud et al., 2019).

Memory is thought to be associated with the medial temporal lobe (MTL) and hippocampus (Schaapsmeerders et al., 2015; Chen et al., 2021), while the hippocampus is the core brain region in the limbic system responsible for the formation and consolidation of memory (Cechova et al., 2020). To date, many studies have focused on memory impairments linked to structural and functional damage in the hippocampus (Zeineh et al., 2003), and magnetic resonance imaging (MRI) studies have highlighted a reduction in hippocampal volume (Dimsdale-Zucker et al., 2018). However, studies have found that the hippocampus comprises several subfields shown to differentially support memory (Wu et al., 2019), and these substructures are more sensitive than the whole in predicting pathological alterations (Marizzoni et al., 2015). Thus, treating the hippocampus as a single homogeneous entity in neuroimaging studies may not be entirely appropriate (Bender et al., 2019). Advances in computational methods and atlases available for anatomical MRI allow the automated segmentation of hippocampal subfields, which helps to better understand the mechanisms of how the hippocampus affects memory. Some studies have applied this kind of technology to confirm that the CA3 and DG are involved in memory encoding and early retrieval. CA1 is primarily responsible for recognition and late retrieval (Vaculik et al., 2019), and the subiculum is active during the recollection of learning episodes (Eldridge et al., 2005). In SIVD related research, Gemmell et al. (2012) and Jokinen et al. (2020) have shown that memory impairments in SIVD are related to hippocampal atrophy, and this relationship was also found in the preclinical phase (Kim et al., 2014). However, the division of the hippocampus has been insufficiently precise in related research work, and few studies have followed substructure volume changes in patients at an early stage, a high prevalence and important target population for prevention of change (Hughes et al., 1982).

In this study, we aimed to evaluate the relationship between changes in hippocampal subfield volume and memory dysfunction in patients with SIVD using structural MRI. We hypothesized that (a) SIVD patients, especially SIVD patients with cognitive impairment (SIVD-CI), might have smaller hippocampal subfield volumes than healthy control (HC) subjects. (b) SIVD-CI patients might have reduced volumes in particular hippocampal subfields compared to SIVD patients with normal cognition (HC-SIVD). (c) Smaller volumes in SIVD patients would be associated with worse memory function.

## Materials and Methods

### Participants

All subjects provided written informed consent after a complete explanation of the procedures involved. In total, 39 patients with cognitive impairment (SIVD-CI group), 38 patients without cognitive impairment (HC-SIVD group), and 41 matched elderly healthy control subjects (HC group) were recruited. SIVD was diagnosed based on the following criteria (Román et al., 2002): (1) WMH defined as extensive deep and periventricular white matter lesions observed in T2-weighted images, as extension caps of more than 10 mm (measured along the axial direction of the anterior horn of the lateral ventricle) or irregular halos (>10 mm, irregular boundary extending to deep white matter), or as diffuse fused high signals (>25 mm, with a less regular boundary) or extensive white matter lesions (borderless diffuse high signals); and (2) lacunar infarction involving multiple lacunas (>5) in the subcortex (including deep gray matter or white matter) with a lesion diameter greater than 3 mm but less than 15 mm.

The patients with SIVD were divided into two groups: those without cognitive deficits (HC-SIVD group) and those with mild cognitive impairment (SIVD-CI group). The inclusion criteria for the HC-SIVD group were as follows: (1) age > 55 years and fulfillment of the diagnostic criteria for SIVD; (2) no recent complaints of cognitive impairment and normal daily life activities; (3) Mini-Mental State Examination (MMSE) score ≥ 26; and (4) Clinical Dementia Rating Scale (CDR) score = 0. The inclusion criteria for the SIVD-CI group were as follows: (1) age > 55 years and fulfillment of the diagnostic criteria for SIVD; (2) participant or caregiver complaints that the patient had experienced cognitive decline in at least one cognitive domain; (3) MMSE score < 26; (4) failure to meet the Diagnostic and Statistical Manual of Mental Disorders, fifth edition (DSM-V) criteria for dementia; and (5) CDR score = 0.5.

The inclusion criteria for the healthy control group were as follows: (1) age > 55, no central nervous system disease, willingness to cooperate with the experimental arrangement, and emotional stability; (2) no other diseases affecting the experimental evaluation; (3) MMSE score > 26; and (4) CDR score = 0.

Exclusion criteria for all participants included a history of craniocerebral trauma, psychiatric or neurological disease, other preexisting brain lesions visible on MRI except for WMH and lacunae, other medical complications, active alcohol or illicit drug use, and pregnancy. This study was approved by the research ethics committees of the Affiliated Hospital of North Sichuan Medical College. The patients/participants provided written informed consent to participate in this study. This research was conducted in accordance with the Declaration of *Helsinki and Istanbul.*

### Neuropsychological Assessment

All individuals underwent an extensive neuropsychological battery. Daily life capacities were assessed by the activities of daily living scale (ADL) and CDR (Hughes et al., 1982). The Hamilton depression rating scale (HAM-D) and Hamilton anxiety rating scale (HAM-A) were applied to exclude those with potential depression and anxiety. Global cognition was measured by the MMSE. The auditory memory test was conducted by the Rey’s Auditory Verbal Learning Test (RAVLT) (Vakil and Blachstein, 1993); the Rey–Osterrieth Complex Figure Test (ROCF) (Shin et al., 2006) was used to evaluate visual memory. The digit span test (DST) and reverse digit span test (R-DST) are simple and effective in assessing working memory.

### Magnetic Resonance Imaging Acquisition

All MR images were acquired on a 3.0T GE Medical Systems MR machine (Discovery MR750, United States) with a standard 32-channel head coil. The parameters of high-resolution 3D-T1 were as follows: repetition time (TR) = 8.3 ms, echo time (TE) = 3.3 ms, flip angle = 15°, field of view (FOV) = 240 mm × 240 mm, matrix = 240 × 240, thickness = 1.0 mm, and no gap. Evaluation of SIVD-related pathology was observed on T2 fluid-attenuated inversion recovery (FLAIR)–weighted images: TR = 8,000 ms, TE = 126 ms, inversion time (TI) = 2,100 ms, thickness = 3 mm, FOV = 240 mm × 240 mm, and matrix = 256 × 192.

### Imaging Analysis

FreeSurfer 6.0^1^ (Iglesias et al., 2015; Roddy et al., 2019) was used for cortical reconstruction and volumetric segmentation. The detailed segmentation process briefly includes T1-weighted image motion correction, affine registration to the Talairach space, B1 field uniformity correction, skull stripping using a hybrid watershed algorithm, automatic volume labeling, segmentation of subcortical white matter and deep gray matter structures (including the hippocampus), subcortical structure filling and pruning, construction of a cortical network, etc. Intracranial volume (ICV) was also calculated.

**FIGURE 1:**
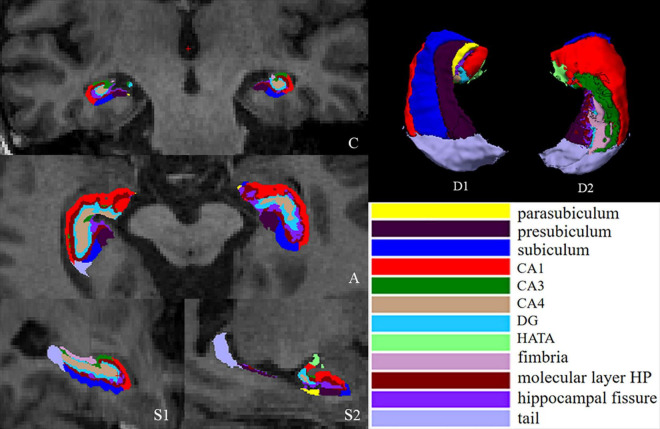
Segmentation of hippocampal subfields. (C) Coronal slice, medial view. (A) Axial slice, medial view. (S1) Sagittal slice, medial view. (S2) Sagittal slice, lateral view. (D1) 3D rendering of segmentation, inferior view. (D2) 3D rendering, anterior view. CA, cornus ammonis; DG, dentate gyrus; HATA, hippocampal amygdalar transition area.

The hippocampus was automatically segmented into 12 subfields, including the tail, CA1, CA3, CA4, dentate gyrus (DG), fimbria, molecular layer HP, hippocampal fissure (cerebrospinal fluid), subiculum, parasubiculum, presubiculum and hippocampal amygdalar transition area (HATA) (Figure 1), in each hemisphere by using the hippocampal subfield module based on a statistical atlas built primarily on ultrahigh-resolution *ex vivo* MRI data in FreeSurfer 6.0. The segmentation algorithm of a single subfield is based on Bayesian inference of image intensity.

Finally, twelve hippocampal subfield volumes were calculated. The scale for the hippocampal structure volumes is related to the total volume of the brain (Vaculik et al., 2019), so the volume of hippocampal subfields can be adjusted by the following formula according to ICV:


$${\text{volume}}_{\text{adjusted}}={\mathrm{volume}}_{\text{raw}}-b\times \left(\text{ICV}-\mathrm{mean}\mathrm{ICV}\right)$$

where b is the regression slope of hippocampal subfield volume on ICV (Chen et al., 2016).

The numbers of lacunar infarcts were counted on T2 FLAIR images. WMH was observed on T2 FLAIR images and assessed by the Fazekas scale (0–6 points for left and right, respectively, up to 12 points; Wardlaw et al., 2013). Evaluation of these two indicators was visually evaluated by two experienced radiologists blinded to the group status.

### Statistical Analysis

IBM SPSS 22.0 (IBM SPSS Inc., Chicago, IL, United States) was used for statistical analysis. The demographic, clinical and neuropsychological data among the SIVD-CI, HC-SIVD and HC groups were compared using one-way analysis of variance (ANOVA), and the chi-square test was used to compare sex distribution. *Post hoc t*-tests were used to detect the differences between each pair of groups. The normality of all volume data was tested by the Kolmogorov–Smirnov test. All statistical tests were two-tailed, and *p* < 0.05 was considered statistically significant.

One-way ANOVA was conducted to test group differences in hippocampal subfield volumes among the three groups with sex, age, education level and ICV as nuisance variables. *Post hoc t*-tests were subsequently applied to detect differences between each pair of groups. Multiple comparisons were corrected using the false discovery rate (FDR) correction approach with *p* < 0.05.

Finally, partial correlation analyses were conducted to examine the relations between these significantly different volumes (between the SIVD-CI and HC-SIVD groups) of hippocampal subfields and scores for auditory, visual, and working memory performance while controlling for age, sex, education years and ICV in SIVD patients.

## Results

### Demographic and Clinical Characteristics

Subject demographic and clinical data are summarized in Table 1. There were no significant differences in sex distribution (*p* = 0.56), age (*p* = 0.73), or educational level (*p* = 0.12). The prevalence of systolic blood pressure (SBP) and hypertension in the SIVD patients (including both the HC-SIVD and SIVD-CI groups) was higher than that in the HC group (*p* < 0.001), and the content of high-density lipoprotein (HDL) was lower than that in the HC group (*p* < 0.001). Among SIVD patients, the prevalence of diabetes in the SIVD-CI group was higher than that in the HC-SIVD group (*p* = 0.01).

**TABLE 1:** Comparisons of demographic and clinical data of the SIVD-CI, HC and HC-SIVD groups.

	SIVD-CI (*n* = 39)	HC (*n* = 41)	HC-SIVD (*n* = 38)	F/χ^2^	*p-*value
**Demographic factors**					
Age (years)	69.72 ± 6.26	68.71 ± 5.99	69.03 ± 4.97	0.32	0.73
Education (years)	9.10 ± 2.00	9.39 ± 2.87	10.26 ± 2.68	2.16	0.12
Sex (M/F)	22/17	21/20	24/14	1.15	0.56
ICV (mm^3^)	1.46 × 10^6^ ± 1.60 × 10^5^	1.41 × 10^6^ ± 1.50 × 10^5^	1.43 × 10^6^ ± 1.21 × 10^5^	1.10	0.34
**Vascular risk factors**					
BMI (kg/m^2^)	23.09 ± 2.93	23.00 ± 1.99	24.26 ± 2.31	3.24	0.04^c^
SBP (mmHg)	148.41 ± 21.74	130.46 ± 14.92	147.84 ± 21.95	10.73	<0.001^a,c^
DBP (mmHg)	79.85 ± 10.78	77.17 ± 9.17	80.97 ± 11.83	1.34	0.23
Blood glucose (mmol/L)	5.98 ± 1.58	6.26 ± 2.71	6.47 ± 2.00	0.51	0.60
Total cholesterol (mmol/L)	4.64 ± 1.45	5.12 ± 1.24	4.22 ± 1.02	5.59	0.005^c^
Triglyceride (mmol/L)	1.62 ± 0.85	1.55 ± 0.77	1.47 ± 0.82	0.48	0.62
HDL (mmol/L)	1.38 ± 0.31	1.63 ± 0.32	1.34 ± 0.30	10.75	<0.001^a,c^
LDL (mmol/L)	2.93 ± 1.16	3.16 ± 1.16	2.50 ± 0.89	3.85	0.02^c^
**History**					
Smoking (Y/N)	30/9	32/9	28/10	0.22	0.90
Drinking (Y/N)	32/7	37/4	27/11	4.81	0.09
Diabetes (Y/N)	32/7	36/5	23/15	9.12	0.01^b,c^
HBP (Y/N)	12/27	34/7	13/25	27.34	<0.001^a,c^
CAD (Y/N)	38/1	41/0	33/3	4.14	0.13
Lacunes	2.74 ± 2.10	0.02 ± 0.16	2.21 ± 2.17	28.00	<0.001^a,c^
WMH	7.33 ± 2.12	0.34 ± 1.54	5.21 ± 2.35	126.21	<0.001^a,b,c^

*Normally distributed variables are expressed as the mean ± SD, and categorical variables are expressed as percentage.*

*SIVD-CI, SIVD patients with cognitive impairment; HC-SIVD, SIVD patients with normal cognition; HC, healthy controls.*

*^a^Significant difference between the SIVD-CI and HC groups (p < 0.05).*

*^b^Significant difference between the SIVD-CI and HC-SIVD groups (p < 0.05).*

*^c^Significant difference between the HC and HC-SIVD groups (p < 0.05).*

*M/F, male/female; ICV, intracranial volume; BMI, body mass index; SBP, systolic blood pressure; DBP, diastolic blood pressure; HDL, high-density lipoprotein; LDL, low-density lipoprotein; HBP, high blood pressure; CAD, coronary artery disease; WMH, white matter hyperintensities.*

Neuropsychological test results including memory tests are listed in Table 2. Significant differences among the three groups were observed on the RAVLT, R-DST and ROCF (*p* < 0.001). The SIVD-CI group performed significantly worse in all memory tests than the HC and HC-SIVD groups except DST (*p* = 0.06), whereas no significant differences were found between the HC and HC-SIVD groups, with the exceptions of RAVLT immediate recall (*p* = 0.001) and ROCF delayed recall (*p* = 0.02) scores.

**TABLE 2:** Results of Neuropsychological Tests in the SIVD-CI, HC and HC-SIVD groups.

	SIVD-CI (*n* = 39)	HC (*n* = 41)	HC-SIVD (*n* = 38)	ANOVA		SIVD-CI vs. HC	SIVD-CI vs. HC-SIVD	HC-SIVD vs. HC
				*F*	*p*	*p*	*p*	*p*
MMSE	22.28 ± 4.42	28.00 ± 1.16	27.90 ± 1.25	56.51	<0.001	<0.001	<0.001	0.70
HAM-A	3.92 ± 1.61	2.98 ± 2.66	3.50 ± 1.77	2.09	0.13	–	–	–
HAM-D	2.82 ± 2.29	2.46 ± 2.15	2.79 ± 2.68	0.28	0.76	–	–	–
**Memory**								
**RAVLT**								
Immediate recall	3.80 ± 2.75	9.27 ± 2.01	7.32 ± 3.02	44.74	<0.001	<0.001	<0.001	0.001
Delayed recall	3.28 ± 2.58	8.56 ± 2.49	7.47 ± 2.85	44.15	<0.001	<0.001	<0.001	0.07
Recognition	13.85 ± 7.54	16.02 ± 4.78	20.87 ± 6.09	12.85	<0.001	<0.001	<0.001	0.13
DST	7.31 ± 1.51	7.88 ± 1.10	8.29 ± 1.27	5.54	0.005	0.06	0.003	0.13
R-DST	2.80 ± 0.89	4.07 ± 0.85	3.97 ± 1.37	17.83	<0.001	<0.001	<0.001	0.70
**ROCF**								
Immediate recall score	25.49 ± 11.22	34.42 ± 2.24	33.24 ± 4.67	18.39	<0.001	<0.001	<0.001	0.16
Delayed recall score	6.31 ± 6.50	16.68 ± 6.08	13.11 ± 7.14	25.53	<0.001	<0.001	<0.001	0.02

*Normally distributed variables are expressed as the mean ± SD.*

*SIVD-CI, SIVD patients with cognitive impairment; HC-SIVD, SIVD patients with normal cognition; HC, healthy controls.*

*MMSE, Mini-Mental State Examination; HAM-A, Hamilton anxiety rating scale; HAM-D, Hamilton depression rating scale.*

*RAVLT, Rey’s Auditory Verbal Learning Test; DST, digit span test; R-DST, reverse digit span test; ROCF, Rey–Osterrieth Complex Figure Test.*

### Hippocampal Subfield Volume Differences Among the Three Groups

**FIGURE 2:**
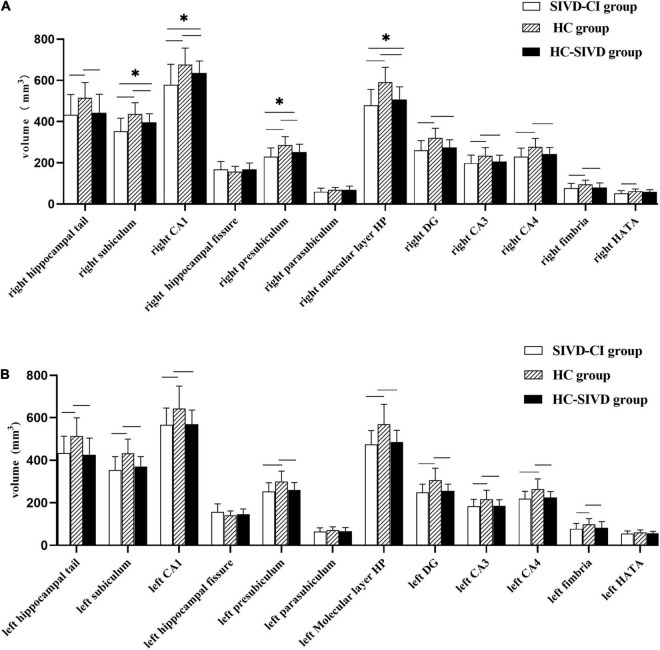
Differences in subfield volumes of right **(A)** and left **(B)** hippocampus among the SIVD-CI, HC-SIVD and HC groups. SIVD-CI, SIVD patients with cognitive impairment; HC-SIVD, SIVD patients with normal cognition; HC, healthy control. CA, cornus ammonis; DG, dentate gyrus; HATA, hippocampal amygdalar transition area. − Significant difference between the SIVD-CI and HC groups, HC-SIVD and HC groups after FDR correction for multiple comparisons. * Significant difference between the SIVD-CI and HC-SIVD groups after FDR correction for multiple comparisons.

**FIGURE 3:**
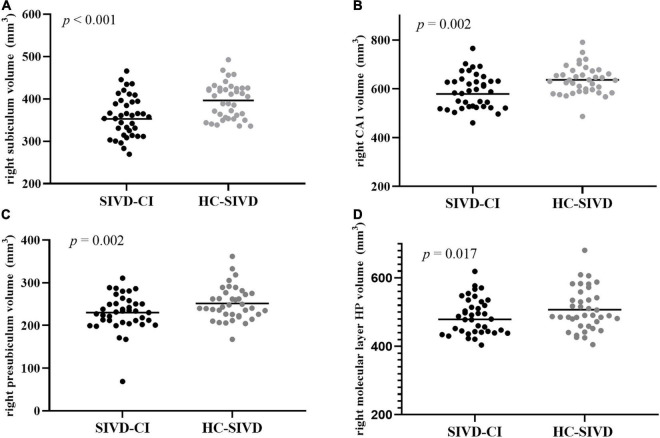
Differences between the SIVD-CI and HC-SIVD groups of hippocampal subfield volumes in the right subiculum **(A)**, right CA1 **(B)**, right presubiculum **(C)**, and right molecular layer HP **(D)**. CA, cornus ammonis.

The Kolmogorov–Smirnov test confirmed that all hippocampal subfield volume data were normally distributed (*p* > 0.05). ANOVA revealed that volume differences were found in each of the subfields of the hippocampus, except the bilateral hippocampal fissure (*p* = 0.366, *p* = 0.086, respectively.) and left parasubiculum (*p* = 0.166) among the SIVD-CI, HC-SIVD, and HC groups (Tables 3, 4 and Figure 2) when age, sex, educational level and ICV were considered as nuisance variables. Then, *post hoc* pairwise comparisons were used to compare the volume changes in the hippocampal subfields between each pair of groups. Compared with HCs, SIVD-CI and HC-SIVD patients showed extensively decreased volumes in the bilateral hippocampal tail, subiculum, CA1, presubiculum, molecular layer HP, DG, CA3, CA4 and fimbria (*p* < 0.05). Compared with the HC-SIVD group, the SIVD-CI group exhibited significant atrophy in the right subiculum (*p* < 0.001), CA1 (*p* = 0.002), presubiculum (*p* = 0.002) and molecular layer HP (*p* = 0.017) (Table 2 and Figures 2, 3) after FDR correction.

**TABLE 3:** Analysis of covariance of the volumes of right hippocampal subfields among the three groups.

	SIVD-CI (*n* = 39)	HC (*n* = 41)	HC-SIVD (*n* = 38)	ANOVA		SIVD-CI vs. HC	SIVD-CI vs. HC-SIVD	HC-SIVD vs. HC
				*F*	*P*	*p*_adjusted_	*p*_adjusted_	*p*_adjusted_
Hippocampal tail	433.27 ± 98.48	515.61 ± 74.21	442.32 ± 90.59	13.232	<0.001	<0.001	0.187	<0.001
Subiculum	352.70 ± 64.11	436.68 ± 54.74	396.62 ± 41.57	30.704	<0.001	<0.001	<0.001	<0.001
CA1	578.89 ± 99.37	676.58 ± 80.45	636.57 ± 57.93	20.051	<0.001	<0.001	0.002	0.004
Hippocampal fissure	168.50 ± 38.16	156.82 ± 25.85	167.48 ± 31.60	1.013	0.366	–	–	–
Presubiculum	230.08 ± 42.26	286.58 ± 40.38	251.34 ± 39.19	25.497	<0.001	<0.001	0.002	<0.001
Parasubiculum	59.46 ± 17.59	68.17 ± 12.35	68.76 ± 17.94	5.459	0.005	0.008	0.058	0.988
Molecular layer HP	479.16 ± 77.10	591.15 ± 72.57	507.43 ± 61.54	36.452	<0.001	<0.001	0.017	<0.001
DG	260.87 ± 46.63	320.13 ± 47.71	275.29 ± 36.20	25.781	<0.001	<0.001	0.058	<0.001
CA3	198.21 ± 39.39	233.81 ± 40.12	205.91 ± 31.02	13.475	<0.001	<0.001	0.197	<0.001
CA4	229.89 ± 41.97	277.68 ± 40.68	242.80 ± 31.66	21.970	<0.001	<0.001	0.054	<0.001
Fimbria	77.28 ± 23.08	95.59 ± 20.00	79.51 ± 23.06	9.883	<0.001	<0.001	0.858	<0.001
HATA	51.65 ± 13.39	62.60 ± 10.38	59.17 ± 10.62	11.585	<0.001	<0.001	0.060	0.070
Whole hippocampus	2951.46 ± 492.45	3564.59 ± 414.66	3165.72 ± 488.25	30.750	<0.001	<0.001	0.004	<0.001

*The volumes (mm^3^) of each subfield of the hippocampus are expressed as the mean ± SD.*

*SIVD-CI, SIVD patients with cognitive impairment; HC-SIVD, SIVD patients with normal cognition; HC, healthy control.*

*CA, cornus ammonis; DG, dentate gyrus; HATA, hippocampal amygdalar transition area.*

*FDR, False discovery rate was used for multiple corrections, and p_adjusted_ was the adjusted p-value between each two groups.*

**TABLE 4:** Analysis of covariance of the volumes of left hippocampal subfields among the three groups.

	SIVD-CI (*n* = 39)	HC (*n* = 41)	HC-SIVD (*n* = 38)	ANOVA		SIVD-CI vs. HC	SIVD-CI vs. HC-SIVD	HC-SIVD vs. HC
				*F*	*p*	*p*_adjusted_	*p*_adjusted_	*p*_adjusted_
Hippocampal tail	433.55 ± 79.55	513.57 ± 86.08	426.11 ± 78.36	16.839	<0.001	<0.001	0.613	<0.001
Subiculum	354.59 ± 62.20	432.25 ± 67.27	370.43 ± 46.80	25.110	<0.001	<0.001	0.084	<0.001
CA1	566.12 ± 80.18	644.29 ± 105.60	569.35 ± 67.69	14.445	<0.001	<0.001	0.441	<0.001
Hippocampal fissure	156.97 ± 37.95	140.38 ± 21.57	146.66 ± 24.10	2.507	0.086	–	–	–
Presubiculum	253.37 ± 41.03	299.96 ± 48.93	259.82 ± 35.38	18.188	<0.001	<0.001	0.208	<0.001
Parasubiculum	65.26 ± 17.15	70.83 ± 16.18	65.58 ± 18.32	1.826	0.166	–	–	–
Molecular layer HP	474.34 ± 65.15	569.98 ± 93.61	485.52 ± 55.42	27.325	<0.001	<0.001	0.137	<0.001
DG	249.43 ± 38.13	306.10 ± 56.41	256.11 ± 31.44	27.219	<0.001	<0.001	0.220	<0.001
CA3	183.76 ± 32.69	216.32 ± 42.85	184.68 ± 29.69	14.293	<0.001	<0.001	0.593	<0.001
CA4	219.55 ± 34.88	263.90 ± 48.64	225.11 ± 28.17	22.188	<0.001	<0.001	0.249	<0.001
Fimbria	77.90 ± 24.94	99.03 ± 26.61	83.25 ± 28.19	7.462	0.001	<0.001	0.880	0.006
HATA	54.83 ± 12.76	60.68 ± 11.97	56.48 ± 8.93	3.749	0.027	0.045	0.502	0.080
Whole hippocampus	2932.70 ± 397.47	3476.89 ± 542.54	2982.42 ± 340.13	25.924	<0.001	<0.001	0.221	<0.001

*The volumes (mm^3^) of each subfield in the hippocampus are expressed as the mean ± SD.*

*SIVD-CI, SIVD patients with cognitive impairment; HC-SIVD, SIVD patients with normal cognition; HC, healthy control.*

*CA, cornus ammonis; DG, dentate gyrus; HATA, hippocampal amygdalar transition area.*

*FDR, False discovery rate was used for multiple correction, and p_adjusted_ was the adjusted p-value between each two groups.*

### Relationships Between Hippocampal Subfield Volumes and Memory Scale Scores in Subcortical Ischemic Vascular Disease Patients

**FIGURE 4:**
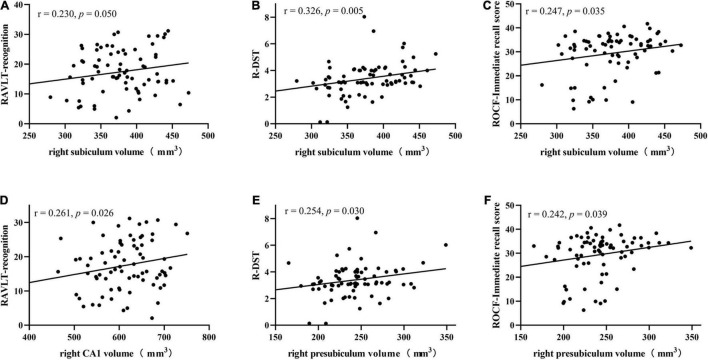
Correlations between right subiculum volumes and RAVLT recognition **(A)**, R-DST **(B)**, and ROCF immediate recall scores **(C)**, right CA1 volumes and RAVLT recognition scores **(D)**, right presubiculum volumes and R-DST **(E)**, ROCF immediate recall scores **(F)** in SIVD patients. RAVLT, Rey’s Auditory Verbal Learning Test; R-DST, reverse digit span test. Volume data are expressed in mm^3^.

As shown in Table 5, right subiculum volumes were positively related to RAVLT word recognition (*r* = 0.230, *p* = 0.050) (Figure 4A), R-DST (*r* = 0.326, *p* = 0.005) (Figure 4B) and ROCF immediate recall (*r* = 0.247, *p* = 0.035) scores (Figure 4C), right CA1 volumes were positively correlated to RAVLT word recognition (*r* = 0.261, *p* = 0.026) (Figure 4D), and right presubiculum volumes showed positive relationships with R-DST (*r* = 0.254, *p* = 0.030) (Figure 4E) and ROCF immediate recall (*r* = 0.242, *p* = 0.039) scores (Figure 4F).

**TABLE 5:** Correlations between memory scores and hippocampal subfield volumes with intergroup differences in SIVD patients.

	Right subiculum		Right CA1		Right presubiculum		Right molecular layer HP	
	*r*	*P*	*r*	*p*	*r*	*p*	*r*	*p*
**RAVLT**								
Immediate recall	0.045	0.707	−0.062	0.605	0.022	0.855	−0.134	0.259
Delayed recall	0.175	0.138	0.064	0.588	0.119	0.318	0.002	0.987
Recognition	0.230	0.050*	0.261	0.026*	0.203	0.086	0.217	0.066
DST	−0.042	0.727	0.011	0.926	0.024	0.839	−0.086	0.472
R-DST	0.326	0.005*	0.208	0.077	0.254	0.030*	0.129	0.278
**ROCF**								
Immediate recall score	0.247	0.035*	0.142	0.231	0.242	0.039*	0.160	0.176
Delayed recall score	0.119	0.315	0.102	0.389	0.163	0.168	0.054	0.649

*CA, cornus ammonis; HATA, hippocampal amygdalar transition area.*

*RAVLT, Rey’s Auditory Verbal Learning Test; DST, digit span test; R-DST, reverse digit span test; ROCF, Rey–Osterrieth Complex Figure Test.*

p-values with “*” indicate significant correlation.

## Discussion

In this study, we assessed alterations in hippocampal subfield volumes in SIVD patients with and without cognitive deficits compared to healthy senior controls. Both the SIVD-CI and HC-SIVD groups showed extensively decreased volumes across hippocampal subfields, including the bilateral hippocampal tail, subiculum, CA1, presubiculum, molecular layer HP, DG, CA3, CA4, and fimbria. In the SIVD subgroup comparison, the SIVD-CI group, compared with the HC-SIVD group, exhibited specific atrophy in the right subiculum, CA1, presubiculum, and molecular layer HP. Moreover, the volumes in the right subiculum were positively correlated with RAVLT word recognition, R-DST and ROCF immediate recall scores, CA1 volumes were positively related to RAVLT word recognition, and the presubiculum was positively correlated with the R-DST and ROCF immediate recall scores in SIVD patients.

### Clinical Indicators and Memory Alterations Among Subcortical Ischemic Vascular Disease Patients and Healthy Controls

It has been confirmed that vascular disease is an important risk factor that promotes cognitive decline (including memory) and is an early expression of dementia (Xu et al., 2019). The main reason may be that SIVD gives rise to insufficient cerebral blood supply, which leads to local tissue ischemia and eventually leads to changes in brain function, especially memory disorders (Broadhouse et al., 2019). In this study, the SIVD-CI patients showed lower scores on verbal (RAVLT) and visual (ROCF) delayed recall tests than the HCs and HC-SIVD patients, consistent with the observations of Gainotti et al. (2008) and Kim et al. (2014). Although they did not find differences between the two groups in the DST and R-DST, a decrease in scores was observed. Lei et al. (2016) also found that the ROCF test scores were decreased in cognitive impairment patients with SIVD. Our study showed that SIVD patients have memory impairments in the early stage that mainly manifest in auditory, visual and working memory.

### Hippocampal Subfield Alterations in Subcortical Ischemic Vascular Disease Patients

Reduced volumes in all hippocampal substructures were found in the SIVD patients, with the exceptions of the bilateral hippocampal fissure and the left parasubiculum. Hippocampal atrophy is an important pathway of cognitive impairment in vascular disease (Yamamoto et al., 2021) and is associated with neurofibrillary tangle deposition and neuronal loss. It is possible that SIVD might have disrupted the connection between the hippocampus and the cortical areas, leading to secondary degeneration and remote injury, eventually resulting in hippocampal atrophy (Goukasian et al., 2019). Han et al. (2020) have shown that even mild vascular diseases can lead to hippocampal atrophy and cognitive decline. The results of Papma et al. (2012) showed that the volume in the bilateral hippocampus in SIVD patients with MCI was smaller than that in healthy controls. Our results also confirmed that the decrease in hippocampal volume is a comprehensive manifestation of decreases in hippocampal subfield volumes. In addition, reductions in hippocampal volume can exist in SIVD patients with normal cognition, which suggests that hippocampal atrophy occurs earlier than memory impairments.

Compared to the whole hippocampus, hippocampal subfields are differentially sensitive to pathological alterations (Marizzoni et al., 2015). Broadhouse et al. (2019, 2020) showed that specific atrophy within the CA1 region and subiculum correlated with cognitive impairment severity and was associated with an increased risk of conversion from MCI to AD. Hanseeuw et al. (2011) found that the subiculum was significantly atrophic in patients with amnestic MCI compared to HCs. In research involving SIVD, Lambert et al. (2018) found that patients with SIVD had atrophy in the left anterior hippocampus before the onset of dementia. Gemmell et al. (2012) observed that hippocampal CA1 and CA2 volumes decreased in patients with vascular dementia. Kim et al. (2014) found atrophy in the CA1 area in MCI patients with SIVD, and atrophy in patients with vascular dementia extended to the CA1 area and subiculum. Li et al. (2016) found that the left subiculum and presubiculum in patients with subcortical ischemic MCI were significantly atrophic compared with those in HCs. Consistent with these results, the SIVD-CI patients presented greater shrinkage in the right subiculum, CA1 and presubiculum than the HC-SIVD patients. Hippocampal subfields are differentially vulnerable to ischemia, as hippocampal interneurons are believed to be more resistant to the effects of ischemia than larger projection neurons (Bartsch et al., 2015). Postmortem investigations of transient global ischemia patients have revealed bilateral damage to the CA1 pyramidal neurons (Bachevalier and Meunier, 1996). CA1 is most susceptible to ischemia (Bender et al., 2013), which is mirrored in delayed neuronal death (Stamenova et al., 2018). Therefore, CA1 atrophy is the earliest and may be an early imaging marker of SIVD. The literature shows that both AD and vascular dementia cases are likely to exhibit volume changes within the CA1 and subiculum (Seo et al., 2010), which may be the progression of SIVD-CI patients, and the degree of atrophy increase with the course of the disease.

Unlike other studies, we also found that the right molecular layer HP was smaller in the HC-SIVD patients. Carey et al. (2019) showed that the volume of the hippocampal molecular layer HP in adults was correlated with both immediate and delayed memory performance, indicating that the decrease in the hippocampal molecular layer HP volume may be related to memory impairments associated with SIVD. In the comparison between the SIVD-CI and HC-SIVD groups, the specific atrophy of hippocampal subfields were observed, the reason may be that CI patients suffer from more severe ischemia, or the progression of the disease leads to further atrophy of the hippocampus (Kim et al., 2014).

Furthermore, significant differences in hippocampal subfields between SIVD-CI and HC-SIVD were limited over the right hippocampus. In some studies, a laterality effect between the left and right hippocampus was detected, as well as functional lateralization; for example, the right hippocampus may be devoted to episodic memories with spatial and visual components, whereas the left hippocampus may be devoted to memories with verbal or linguistic components (Rolls, 2016). However, left hippocampal atrophy is found to be more evidently related to poorer memory function. The reason we are observing a right-sided and not left-sided association is not clear, may be related to different samples and measurement methods.

### Relationship Between Hippocampal Subfield Alterations and Memory Features

Both hippocampal abnormalities and deficits in episodic memory have been consistently reported in cerebrovascular diseases (Powell et al., 2018). Several recent studies have indicated that there is a relationship between hippocampal subfield volumes and verbal memory, including the subiculum with immediate memory and the CA1 area, subiculum, and presubiculum with delayed memory (Carey et al., 2019). In this study, right CA1 volumes in SIVD patients were positively correlated with word recognition scores on the RAVLT, which was similar to the study of Broadhouse et al. (2019), who found that CA1 region volumes in MCI patients were positively correlated with delayed memory scores on the RAVLT. The CA region encodes verbal memory (Hao et al., 2020), and previous studies have shown that CA1 volumes and delayed memory performance are related to the progression of MCI (Carey et al., 2019). Our results showed that a smaller right subiculum corresponded to worse RAVLT word recognition and ROCF immediate recall memory performance in SIVD patients, similar to the results of Orbo et al. (2018). They found that volumes in the bilateral hippocampal subiculum were positively correlated with the immediate memory and delayed memory scores on the RAVLT in patients with cardiac arrest. As the main output from the hippocampus, the subiculum plays an important role in memory consolidation and is related to delayed memory (Ogawa et al., 2019). Different from their findings, we found that the volume of CA1 and subiculum was related to the recognition of RAVLT, not delayed memory. Recognition additionally draws on familiarity (Argyropoulos et al., 2019), while delayed recall is a vivid, contextually rich memory. The results of this study show that the volume of some hippocampal subregion is also play a part in familiarity, and there is a complex correlation between hippocampus and memory. Volume of the presubiculum was found to be positively correlated with immediate recall scores on the ROCF in our study. As a relay station in the hippocampal neuronal circuit, the presubiculum receives inputs from multiple cortical regions (Li et al., 2016) and has been associated with scene recognition and visuospatial processing (Roddy et al., 2019). In this study, the volumes of the right subiculum and presubiculum were positively correlated with R-DST scores, indicating that reductions in the volumes of the subiculum and presubiculum were related to decreases in working memory in patients with SIVD. Therefore, the results of this study suggested that the reduced volumes in the CA1, subiculum and presubiculum are related to memory impairments in auditory delayed memory, working memory and visual immediate memory in those with SIVD. This shows that the memory impairment may be caused by a reduction in subfield volume, and different volume changes correspond to different types of memory disorders. Evaluating the volume changes may be helpful to understand the mechanism of memory impairment in SIVD patients and for subsequent diagnosis and treatment.

## Limitations

We should point out that our study had some limitations. First, susceptibility-weighted imaging (SWI) was not used to evaluate the microbleeds in the patients, and we did not use quantitative analysis to evaluate the volume of WMH, as their distribution and range are of greater significance in analyzing the mechanism of SIVD. Future studies should include these additional assessments. Second, this study did not include SIVD patients with dementia stage; thus, the difference in hippocampal volumes between cognitive impairment patients and vascular dementia patients could not be evaluated. Finally, this is a cross-sectional study, but evidence shows that the progression of hippocampal atrophy is related to the progression of SIVD and is different from the volume changes in hippocampal subfields in the process of AD. Therefore, longitudinal follow-up of SIVD patients to observe the possible changes in hippocampal morphology with the progression of SIVD will address this limitation.

## Conclusion

The current study found reductions in most hippocampal subfield volumes in SIVD-CI and HC-SIVD patients compared to healthy controls, and SIVD-CI patients have atrophy in specific subfields of the hippocampus. Our results indicate that SIVD patients’ memory impairments occur at an early stage and are associated with reduced volume in particular hippocampal subfields.

## Data Availability Statement

The original contributions presented in the study are included in the article/supplementary material, further inquiries can be directed to the corresponding author/s.

## Ethics Statement

The studies involving human participants were reviewed and approved by the Research Ethics Committees of the Affiliated Hospital of North Sichuan Medical College. The patients/participants provided their written informed consent to participate in this study.

## Author Contributions

MH, YnL, and LC contributed equally to the experiments, data analysis, writing and revising the manuscript. LZ, YjL, and TL contributed to performing the experiments and data analysis. WY and JS contributed to the data collection. All authors contributed to the article and approved the submitted version.

## Conflict of Interest

The authors declare that the research was conducted in the absence of any commercial or financial relationships that could be construed as a potential conflict of interest.

## Publisher’s Note

All claims expressed in this article are solely those of the authors and do not necessarily represent those of their affiliated organizations, or those of the publisher, the editors and the reviewers. Any product that may be evaluated in this article, or claim that may be made by its manufacturer, is not guaranteed or endorsed by the publisher.

## Footnotes

1
http://surfer.nmr.mgh.harvard.edu
